# Prevalence and associated factors of prediabetes in adult East African population: A systematic review and meta-analysis

**DOI:** 10.1016/j.heliyon.2023.e21286

**Published:** 2023-10-20

**Authors:** Daniel Asmelash, Getachew Mesfin Bambo, Samuel Sahile, Yemane Asmelash

**Affiliations:** a Department of Medical Laboratory Science, College of Medicine and Health Sciences, Mizan-Tepi University, Mizan-Aman, Ethiopia; b Department of Statistics, College of Natural and Computational Science, Aksum University, Aksum, Ethiopia

**Keywords:** Prevalence, Associated factors, Prediabetes, Meta-analysis, Systematic review, East Africa

## Abstract

**Introduction:**

Diabetes mellitus is a major public health problem with serious consequences, and more than three-fourths of diabetes live in low- and middle-income countries. According to a recent study, people with prediabetes have nearly six times the risk of developing diabetes than those with normal glucose levels. However, due to the inconsistency and absence of representative data, this study aimed to estimate the prevalence of prediabetes and its associated factors in the adult East African population.

**Methods:**

Databases were systematically searched for articles published between January 1, 2013, and December 30, 2022. All observational community-based studies that reported prediabetes prevalence and/or associated factors in adult East African populations were included in the meta-analyses. Three authors independently extracted all required data using the Excel data extraction format and analyzed using Stata™ Version 11. An I2 test was conducted to determine significant heterogeneity. Finally, a random effects model was used to determine the overall prevalence of prediabetes and its associated factors. The study was registered with Prospero number CRD42023389745.

**Results:**

The search strategy identified 267 articles. After screening for full-text review, twenty-one articles were included in the final analysis. The overall prevalence of prediabetes was 12.58 % (95 % CI:10.30, 14.86 %) in the adult East African population. Furthermore, the subgroup analysis revealed that prediabetes in the urban population 20 % (95 % CI: 1.60, 38.37) was twice as prevalent as in rural 10.0 % (95 % CI: 5.52, 14.48) populations. The prevalence of prediabetes by the ADA diagnostic criteria was 21.45 % (95 % CI: 15.54, 27.35) three times higher than the WHO 7.20 % (95 % CI: 5.70, 8.69). Moreover, prediabetes was significantly associated with old age (OR = 1.64, 95 %, CI: 1.07, 2.53), hypertension (OR = 2.43, 95 %, CI: 1.02–5.79), obesity and overweight (OR = 1.70, 95 %, CI: 1.09,2.65).

**Conclusion:**

This study showed a high prevalence of prediabetes, which was significantly associated with old age, hypertension, and high BMI. This study suggests that health policymakers should pay attention to the prevention and control strategies that is targeted at those with obesity, hypertension, and old age.

## 1 Introduction

Diabetes mellitus (DM) is a major public health problem that has serious consequences. According to recent statistics, 537 million people aged 20–79 years have diabetes worldwide, with more than three out of four adults living in low- and middle-income countries [1,2]. In addition, the number of people with diabetes in Africa is 24 million, with an expected 129 % increase by 2045, the highest increase in any region in the IDF 2021 prediction [1].

Furthermore, the regional prevalence of pre-diabetes in Africa was estimated to be 7.8 % (40.9 million) in 2021 and is projected to reach 8.0 % (84.7 million) by 2030. A high prevalence of diabetes in low-income countries, particularly in East Africa, imposes additional costs on households already burdened by communicable diseases, extreme drought, conflict, and poor health systems [1,3,4].

Prediabetes, also known as impaired fasting glucose (IFG), is a transitional state between normal blood glucose level and diabetes. The American Diabetes Association (ADA) defines this condition as intermediate hyperglycemia that occurs between normal glucose tolerance and type 2 diabetes mellitus (T2DM) [5]**.** Prediabetes was defined by the World Health Organization (WHO) in 1999 as a fasting plasma glucose (FPG) level of 110–125 mg/dL (6.1–6.9 mmol/L). Hence, the ADA chose 100 mg/dL (5.6 mmol/L) as the lower cutoff point [6].

In addition, glycated hemoglobin (HbA1c) testing has a higher sensitivity than FPG for detecting prediabetes and diabetes. One study found that only 59 % of patients diagnosed with diabetes based on HbA1c criteria were detected using the FPG test. This has a significant impact on patient diagnosis, cost, burden, and treatment. Despite the differences in prediabetes definitions and screening criteria, estimates suggest that the number of individuals affected by prediabetes is rapidly increasing worldwide [7].

According to a recent meta-analysis of studies, pre-diabetic individuals develop diabetes 6 times more likely than normoglycemic individuals [8]. Nearly one-third of adults with prediabetes are between the ages of 20 and 39, implying that they will spend many years at high risk of developing T2DM and other health problems [9,10].

Most T2DM patients pass through a 'pre-diabetes' transition stage for several years, which provides an opportunity to identify them and promote timely prevention [6,11]. Most people are unaware of the prediabetes phase until they experience complications of diabetes. The risk of T2DM can be reduced by adopting a healthy lifestyle, such as a healthy dietary pattern, physical activity, and obesity management. Intervention projects have reported decreased diabetes development in prediabetic individuals through treatment and lifestyle interventions [12,13]**.**

Common determining factors of prediabetes in low- and middle-income countries include globalization, unhealthy lifestyles, lack of awareness, and limitations in intervention programs. Excess sugar can promote weight gain and thus prediabetes through extra calories, but has no unique diabetogenic effect at physiological levels [14]. Recent research has revealed that some people with prediabetes develop long-term DM complications, including microvascular and macrovascular complications [15].

Furthermore, HbA1c is a better predictor of diabetic microvascular complications, such as diabetic retinopathy, nephropathy, and neuropathy than FPG. Since microvascular complications worsen patient morbidity, early detection and prevention are critical components of patient care [16]. The ADA now recommends that adults without risk factors be screened for prediabetes and type 2 diabetes, starting at the age of 35 [17].

The prevalence of prediabetes varies significantly in East African populations, ranging from 2.0 to 43.2 %, which shows great variation across different test methods, diagnostic criteria and residential settings [2,18].

Prediabetes is an early warning to prevent the development of diabetes and diabetes-related problems in the future. As a result of its alarming prevalence, inconsistency, and lack of representative data, this study sought to estimate the pooled prevalence of prediabetes and its associated factors in the adult East African population. The findings of this study provide evidence to health policymakers regarding the prevention and management of prediabetes. In addition, it can be used to predict future epidemiological and economic burdens associated with diabetes in East Africans.

## 2 Methods

### 2.1 Search strategy and identification of articles

We searched for articles which reported the prevalence of prediabetes and its associated factors in the East African population. We searched for articles from the Web of Science, Cochrane Library, PubMed, Worldwide Science databases, and gray literature. To avoid duplication, we searched for systematic reviews and meta-analyses of this topic. The keywords used to find studies were “prevalence,” “magnitude,” “burden” “associated factors,” “determinant factors,” “prediabetes,” “impaired fasting glucose” and “Each of East Africa countries.” The selected articles were retrieved and managed using the Endnote X22 software. A review of studies published between 2013 and 2022 was considered to provide insights into the current magnitude of the problem in the region.

Three authors (DA, YA, and GMB) independently searched the articles, and any conflicts between their findings were resolved by discussion and consensus in the presence of the fourth author (SS). Microsoft Excel spreadsheets were used to extract data from the included articles, which were then exported to Stata™ Version 11 for analysis. This study followed the Preferred Reporting Items for Systematic Reviews and Meta-Analyses (PRISMA) [19].

### 2.2 Research question

• What is the pooled prevalence of pre-diabetes and its difference in prevalence based on laboratory test methods, diagnosis criteria, and residence place of the population?
• What are the factors associated with pre-diabetes in the adult East African population?

### 2.3 Objectives

• To determine the pooled prevalence of pre-diabetes in the adult East African population
• To determine the pooled prevalence of pre-diabetes based on laboratory test methods, diagnosis criteria and residence place in the adult East African population
• To determine the associated factors of pre-diabetes in adult East African population

### 2.4 Inclusion and exclusion criteria

All published observational community-based studies conducted on the adult East African population that reported the prevalence of prediabetes and its associated factors were considered. In addition, studies published in peer‐reviewed journals and in English between January 01, 2013, and December 30, 2022, were included.

In addition, institution-based studies, studies not conducted on the adult population, and studies conducted before 2013 were excluded. In addition, the study also excluded other types of studies, such as reviews, reports, and articles with incomplete information. Observational studies without defined population characteristics were excluded.

### 2.5 Outcome measures

The primary outcome of this study was the prevalence of prediabetes and its associated factors among East African adults. Associated factors included advanced age, body mass index (BMI), dyslipidemia, hypertension, and a family history of diabetes. Data collection tools were developed for the included studies.

### 2.6 Data extraction and quality assessment

Data were extracted from the full article using Microsoft Excel data extraction format. The authors name, publication year, study country, sample size, study design, study residence, diagnostic criteria, testing method, prevalence, odds ratio, and 2 × 2 contingency tables were extracted from the original study. Any disagreement between the three authors during collection and inclusion was resolved through discussion and consensus in the presence of the fourth author. Any missing or incomplete data in the published study were requested via e-mail on at least two occasions. Endnote Version X22 software was used to manage references and remove duplicate articles. The quality of the studies was assessed using the Newcastle-Ottawa Scale [20]. The study quality was rated as low bias, moderate bias, and high bias with ≤3 points, 3–6 points and ≥7 points, respectively. Based on the Newcastle-Ottawa scale, 16 of the 21 articles had a low risk of bias and five had a moderate risk of bias (Table 1). The included studies with low and moderate risks of bias could enhance the validity of the study. In addition, the protocol for this systematic review and meta-analysis was registered with PROSPERO registration number CRD42023389745.

**Table 1:** Characteristics of studies that reported prediabetes prevalence and associated factors in adult East African Population, 2023.

study	year	Sample size	Prediabetes (n)	Residence	test method	Criteria	Country	Quality Assessment (Points)
Mayega RW et al.	2013	1497	129	Rural	FPG	WHO	uganda	7 points
Mandha J et al.	2015	561	14	Rural	FPG	WHO	tanzania	8 points
Seifu W et al.	2015	4371	140	Rural	FPG	WHO	Ethiopia	8 points
Bahendeka S et al.	2016	3689	74	Urban&Rural	FPG	WHO	Uganda	8 points
Chiwanga FS et al.	2016	586	81	Urban&Rural	FPG	WHO	Uganda& Tanzania	6 points
Stanifer JW et al.	2016	481	84	Urban&Rural	HbA1C	WHO	tanzania	8 points
Tesfaye T et al.	2016	936	75	Urban&Rural	FPG	WHO	Ethiopia	7 points
Ludwig C et al.	2017	382	50	Urban	FPG	WHO	tanzania	8 points
Worede A et al.	2017	392	47	Rural	FPG	ADA	Ethiopia	6 points
Aynalem SB et al.	2018	402	64	Urban&Rural	FPG	ADA	Ethiopia	6 points
Gebreyes YF et al.	2018	9141	832	Urban&Rural	FPG	ADA	Ethiopia	8 points
Mohamed SF et al.	2018	4164	130	Urban&Rural	FPG	WHO	Kenya	8 points
Ploth DW et al.	2018	739	190	Rural	HbA1C	ADA	tanzania	8 points
Aramo C et al.	2019	130	5	Urban&Rural	FPG	WHO	Uganda	6 points
Endris T et al.	2019	587	100	Urban&Rural	FPG	ADA	Ethiopia	8 points
He Y et al.	2020	1372	515	Urban	HbA1C	ADA	Ethiopia	8 points
Wolde HF et al.	2020	773	72	Urban	FPG	WHO	Ethiopia	8 points
Ampeire IP et al.	2022	370	34	Rural	FPG	WHO	Uganda	6 points
Bavuma CM et al.	2022	7240	406	Urban&Rural	FPG	WHO	rwanda	7 points
Teshome AA et al.	2022	324	140	Urban&Rural	FPG	ADA	Ethiopia	7 points
Eltom MA et al.	2018	5242	678	Urban&Rural	FPG	ADA	sudan	8 points

### 2.7 Statistical analysis

Stata™ version 11 statistical software was used for analysis. The inverse variance index (I2) was used to assess the heterogeneity [21] in the included studies. I^2^ values of 0–25 %, 26–75 %, 76–100 % indicate low, moderate, and substantial heterogeneity, respectively. The data were presented using a graphs, tables and forest plots. To identify the possible source of heterogeneity, a univariate meta-regression model was conducted based on the publication year of the studies. Egger's and Begg's tests were also applied to assess publication bias. To reduce the heterogeneity of the study, subgroup analysis was conducted based on residential location, diagnostic criteria, and test methods for prediabetes. The prevalence of prediabetes and odds ratio (OR) of the associated factors, together with the 95 % confidence interval (CI) were presented using a forest plot. Cohen's coefficient was used to assess inter-rater agreement among the investigators involved in study selection and data extraction [22].

## 3 Results

### 3.1 Exploration of articles

**Fig. 1:**
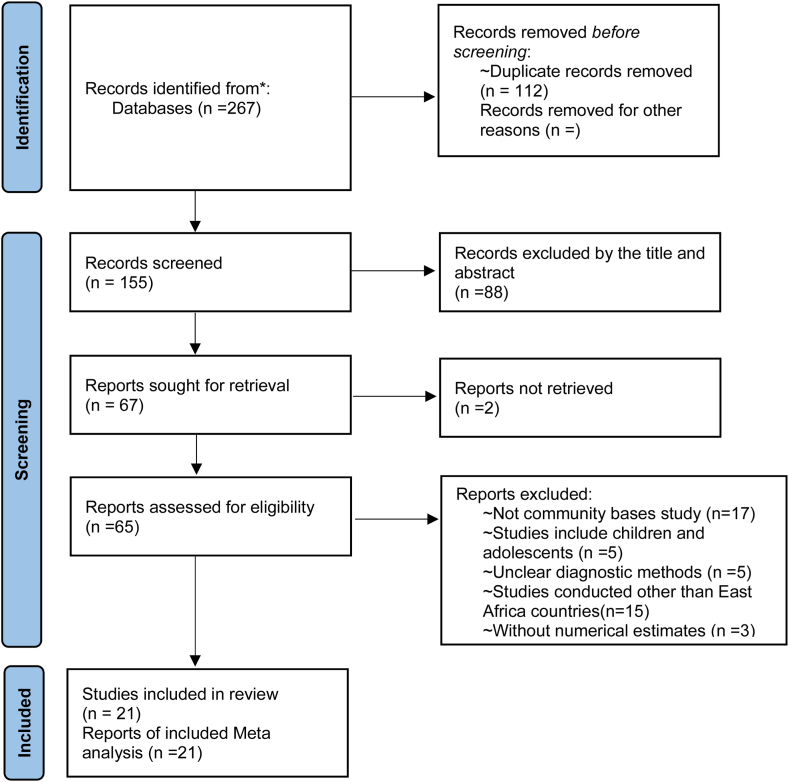
PRISMA 2020 flow diagram for new systematic review diagram of article search and study selection.

We found 267 articles published in various databases. A total of 112 articles were excluded owing to duplication, and 88 articles were excluded based on the title and abstract. Following the review of 65 full texts, the systematic review and meta-analysis included 21 articles that met the inclusion criteria and had high-quality scores. (Fig. 1).

### 3.2 Characteristics of the original articles

A total of 43,379 study participants were used to estimate the pooled prevalence of prediabetes in the adult East African population. The sample sizes of the studies ranged from 130 [23] to 9141 [23]. The articles included in the study were conducted in six countries in the East African region. Among these, the lowest and highest prevalence of prediabetes were reported in theUganda national survey (2 %) [18] and Woreta, Ethiopia (43.2 %) [2], respectively. Only three of the 21 included studies used the HbA1c test method [[24], [25], [26]], and three were conducted in urban areas [[26], [27], [28]]. (Table 1).

### 3.3 Data management and analysis

The overall pooled prevalence of prediabetes was 12.58 % (95%CI:10.30–14.86). Due to the presence of significant heterogeneity between studies ((I2 = 99 %, p < 0.001), we employed a random effect meta-analysis model to estimate the pooled prevalence of prediabetes. The included studies did not exhibit heterogeneity with regard to factors associated with prediabetes (age (I2 = 0 %, p = 0.024), hypertension (I2 = 0 %, p = 0.045) and BMI (I2 = 0 %, p = 0.02)).

**Fig. 2:**
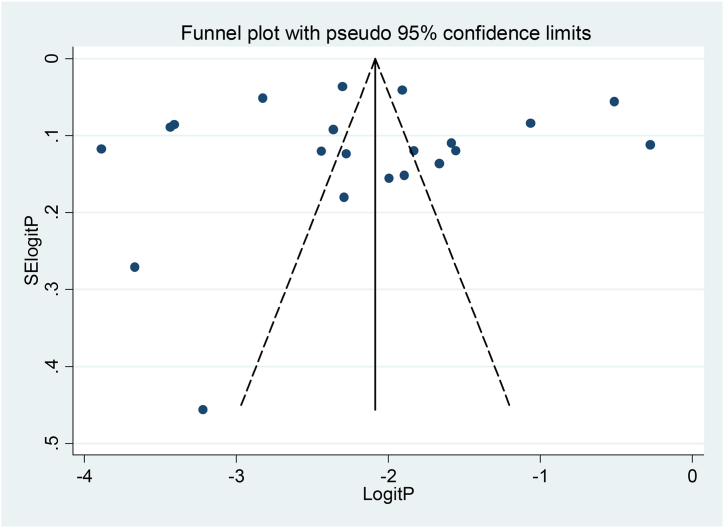
Funnel plot of pooled prediabetes prevalence among adult East African population, 2023.

The funnel plot was visually inspected and no publication bias was observed. In addition, the Egger (*P* = 0.802) and Begg (*P* = 0.695) test values indicated no significant publication bias. In addition, a meta-regression model was conducted on the publication year to detect possible sources of heterogeneity, which was not statistically significant. Moreover, **s**ensitivity analysis was conducted, and the findings revealed no strong evidence for the effect of a single study on the overall pooled result. In addition, a sub-group analysis was performed to identify the sources of heterogeneity. (Fig. 2) (Table 2).

**Table 2:** Egger test for publication bias assessment.

Std_Eff	Coef.	Std. Err.	t	P > t	[95 % Conf. Interval]
slope	−2.00056	.3807387	−5.25	0.000	−2.797458–1.203667
bias	−1.21006	4.749877	−0.25	0.802	−11.15167 8.731542

### 3.4 Prevalence of prediabetes

**Fig. 3:**
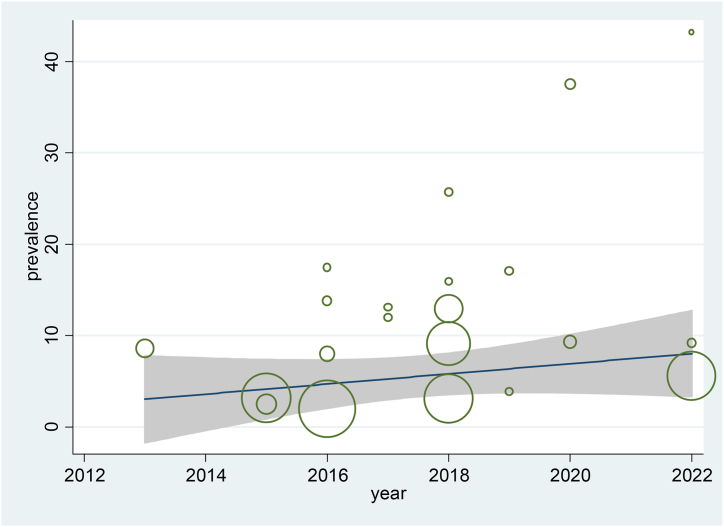
Meta-regression plot showing the trend of prediabetes prevalence in East Africa population over ten years.

The pooled prevalence of prediabetes in the East African population was 12.58 % (95%CI:10.30, 14.86 %). The studies showed significant heterogeneity (I2 = 99 %, p0.001). However, in univariate meta-regression analysis (p = 0.069), publication year was not associated with heterogeneity. In addition, sensitivity analysis revealed that the prevalence of prediabetes ranged from 10.12 % (95 % CI, 6.94%–14.77 %) to 12.2 % (95 % CI, 8.32%–17.76 %). The findings revealed that a single study estimate was closer to the pooled estimate, implying that there was no effect of a single study on the overall pooled result (Supplementary file). In addition, trend analysis showed no significant increase in the prevalence of prediabetes between 2013 and 2022 in the East African population (p value = 0.069) (Fig. 3 and Supplementary file).

### 3.5 Subgroup analysis

**Fig. 4:**
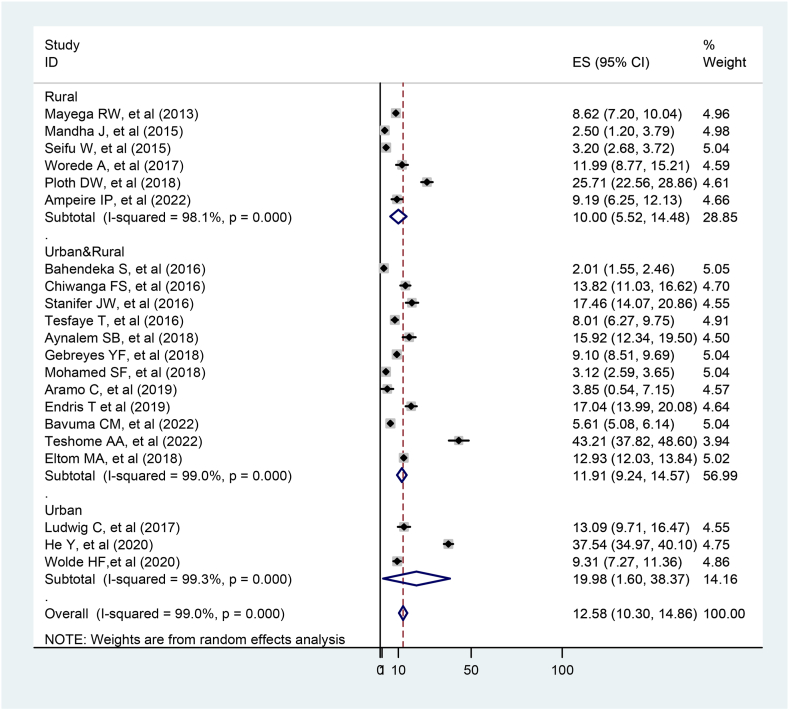
Subgroup analysis on prediabetes prevalence by residence in the adult East African population, 2023.

The prevalence of prediabetes was determined on the basis of the residential locations of the studies. Accordingly, the subgroup analysis revealed that prediabetes in the urban population 19.98 % (95 % CI: 1.60, 38.37) was twice as prevalent as in rural populations 10.0 % (95 % CI: 5.52, 14.48) (P < 0.001) (Fig. 4).

**Fig. 5:**
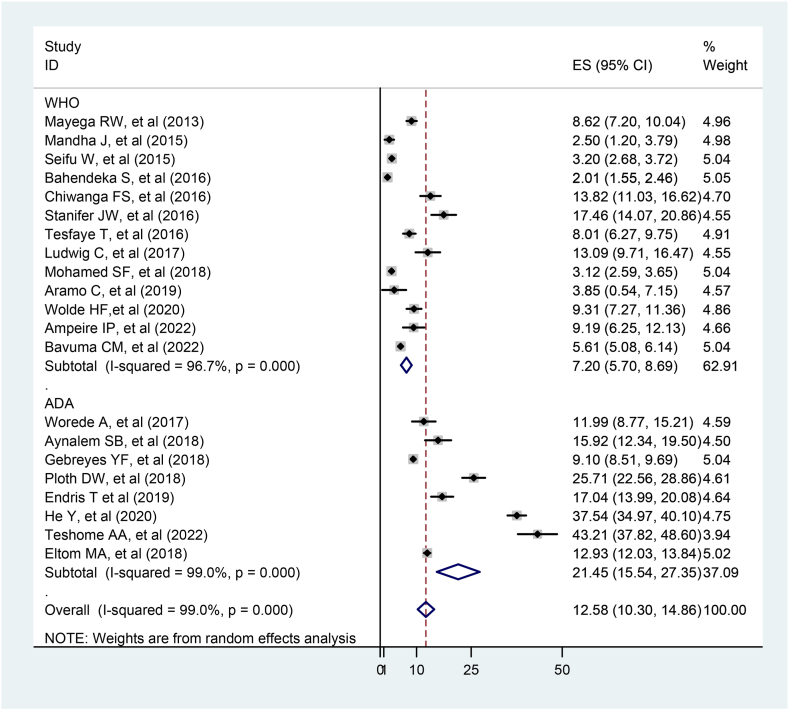
Subgroup analysis of prediabetes prevalence by diagnostic criteria among adult East African population, 2023.

In addition, the prevalence of prediabetes was determined based on the test method and the diagnostic criteria used in the study. As a result, the prevalence of prediabetes according to the ADA diagnostic criteria was 21.45 % (95 % CI: 15.54, 27.35), three times higher than the WHO 7.20 % (95 % CI: 5.70, 8.69) (P < 0.001) (Fig. 5).

**Fig. 6:**
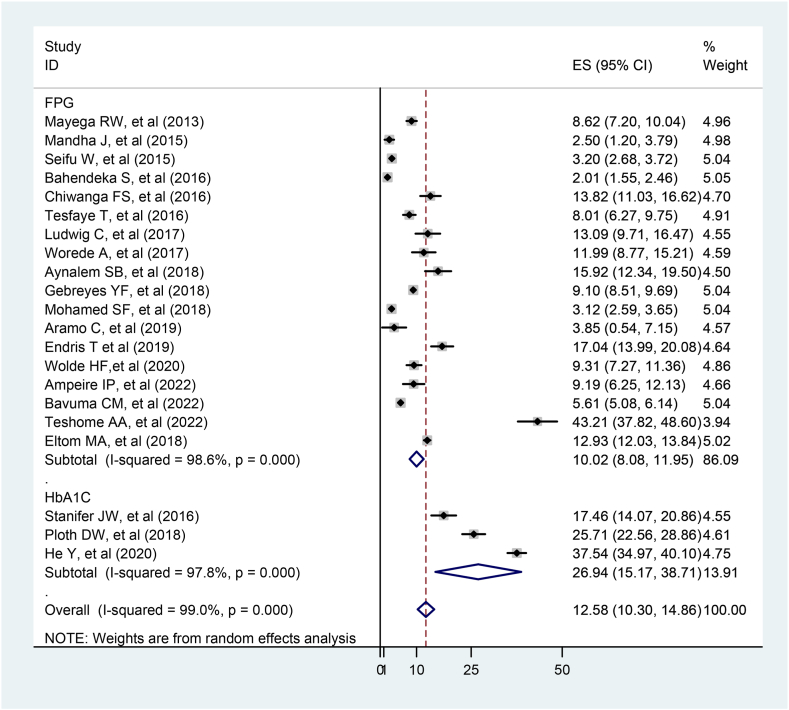
Subgroup analysis of prediabetes prevalence by test methods among adults in the East African population, 2023.

Furthermore, the pooled prevalence of prediabetes using the FPG test method was 10.02 % (95 % CI:8.08, 11.95), which was significantly lower than that using the HbA1c test method 26.94 % (95 % CI: 15.17, 38.71) (P0.001) (Fig. 6).

### 3.6 Factors associated with a prediabetes in East Africa population

#### 3.6.1 Association between prediabetes and old age

**Fig. 7:**
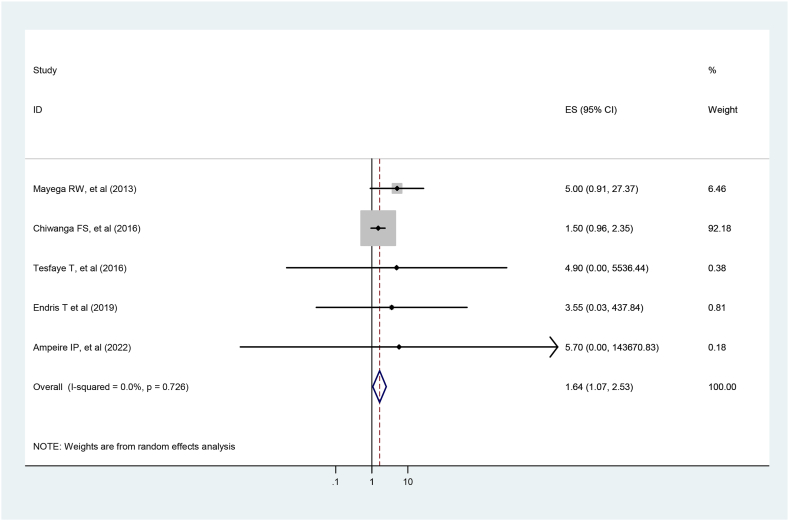
Forest plot for the association between old age and prediabetes.

Five studies were included to reveal the association between prediabetes and old age [3,[29], [30], [31], [32]]. The finding showed that the odds of developing prediabetes was 1.64 times more likely among older adults than younger adults in the 18–24 age group (OR = 1.64, 95 %, CI: 1.067–2.53) (I2 <0.001 %, p = 0.024). (Fig. 7).

#### 3.6.2 Association between prediabetes and hypertension

**Fig. 8:**
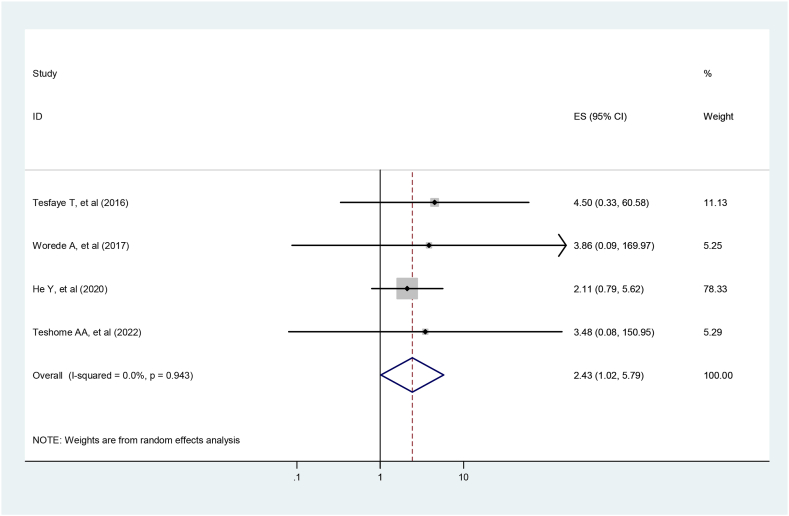
Forest plot for the association between hypertension and prediabetes.

Four studies were included to identify the association between prediabetes and hypertension [2,26,31,33]. The pooled study findings showed that hypertension was significantly associated with prediabetes. Hypertensive individuals were 2.43 times more likely to develop prediabetes than non-hypertensive individuals (OR = 2.43, 95 %, CI: 1.022–5.792). The studies did not show heterogeneity (I2 <0.001 %, *P* = 0.045) (Fig. 8).

#### 3.6.3 Association between prediabetes and BMI

**Fig. 9:**
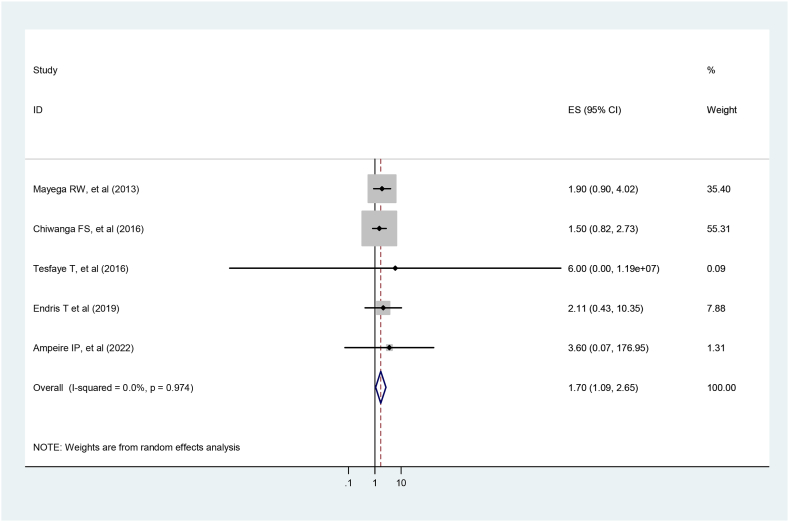
Forest plot for the association between obesity and prediabetes.

The study included five studies that found an association between prediabetes and BMI [3,[29], [30], [31], [32]]. Obese and overweight individuals were 1.7 times more likely to develop prediabetes than normal-weight individuals (OR = 1.7, 95 %, CI: 1.086–2.65). And there was no heterogeneity among the included studies (I2 <0.001 %, *P* = 0.02) (Fig. 9).

## 4 Discussion

This systematic review and meta-analysis was conducted at the subregional level to estimate the prevalence of prediabetes and its associated factors in the adult East African population based on 10 years of data published from 2013 to 2022. This study will contribute to the development of public health strategies to prevent type 2 diabetes through early lifestyle interventions.

The lowest and highest prediabetes prevalence rates were reported in the Uganda National Survey (2 %) [18] and Woreta, Ethiopia (43.2 %) [2], respectively. This may be because the Ugandan study used ADA classification criteria, while the others used WHO classification criteria. This is because the ADA criteria use a lower FBS cutoff point than the latter, resulting in a higher chance of detecting prediabetes. Furthermore, the large differences in sample sizes between these two studies could be the reason for the significant differences in the prevalence of prediabetes.

Generally, the pooled prevalence of prediabetes in the adult East African population was 12.58 % (95%CI:10.30, 14.86 %). The current sub-regional prevalence of prediabetes was higher than that of Africa's regional estimate published by the IDF 2021 Atlas (7.8 %). In addition, this finding was higher than that of community-based studies conducted in Jeddah, Saudi Arabia (10.2 %) [34] and Malaysia (10.1 %) [35]. There is a possibility that the rising prevalence of prediabetes and the predicted epidemic in developing countries may contribute to this.

However, the current prevalence of prediabetes was lower than that reported in community-based studies conducted in the USA [36], China (19 %) [37], Iran (30.29 %) [38], Zhejiang Province, China (18.89 %) [39], and Mexico (47·1 %) [40]. Disparities may occur because of differences in sociodemographic characteristics, sample size, testing methods, diagnostic criteria, and study time.

The prevalence of prediabetes in the rural adult East African population was 10.0 % (95%CI: 5.52, 14.4), which was significantly lower than that in the urban population 19.98 % (95 % CI: 1.60, 38.37) (*P* < 0.001). This finding is supported by studies conducted in Myanmar [41]. Urbanization causes nutritional changes, with an increase in sugar and fat consumption, as well as a sedentary lifestyle. The net result is an epidemiological shift toward higher rates of obesity and diabetes [42].

In addition, the subgroup analysis revealed that the prevalence of prediabetes using ADA diagnostic criteria was 21.45 % (95 % CI: 15.54, 27.35) significantly higher than WHO criteria 7.20 % (95 % CI: 5.70, 8.69) (P < 0.001). This finding is supported by studies conducted in Namibia, West Africa [43]. The higher pooled prevalence of prediabetes under the ADA criteria is due to the fact that the ADA criteria use a lower FBS level than the WHO criteria, resulting in an increased number of people being diagnosed with prediabetes [8].

Moreover, subgroup analysis revealed the prevalence of prediabetes using HbA1c test method 26.94 % (95%CI:15.17, 38.71) was significantly higher compared to FPG test method 10.02 % (95%CI: 8.08, 11.95) (P < 0.001). This could be because HbA1c was used to classify prediabetes, which is far more accurate than the FPG test method [44].

In the current study, old age, residential location, and obesity were identified as factors associated with prediabetes among the adult East African population. Older age groups were 1.64 times more likely to be prediabetic than younger age groups. This result is supported by studies conducted in Malaysia [35], Iran [38], sub-Saharan Africa [45] and Zhejiang, China [39]. Aging is linked to increased adiposity and reduced muscle mass because of the commonly observed decrease in physical activity, which can lead to insulin resistance [34,46].

In addition, the odds of prediabetes among hypertensive individuals were 2.4 times higher compared to normotensive individuals. This is supported by community-based studies in Thailand [44], Zhejiang, China [39], Iran [38], and sub-Saharan Africa [45]. High blood pressure is associated with oxidative stress, immune system activation, inflammation, and blood vessel thickening, which may increase the risk of diabetes [47].

Finally, obesity was found to be a significant factor associated with prediabetes in an adult East African population. Those who were obese and overweight were 1.7 times more likely to experience prediabetes in the adult East African population than those who had a normal BMI. This finding was consistent with studies from Saudi Arabia [34], Namibia, West Africa [43], Iran [38], and Sub-Saharan Africa [45]. Obesity is the strongest predictor of an increased risk of T2DM [34], and because it is a modifiable factor, special consideration should be given to future diabetes intervention programs for the adult East African population.

However, in this study, dyslipidemia was not found to be a significant associated factor for prediabetes in the adult East African population. On the other hand, this result was inconsistent with studies conducted in Thailand [44] and Bangladesh [48]**.**

Therefore, early detection of prediabetes could provide the chance to implement lifestyle interventions as early as possible, potentially preventing diabetes and improving prognosis and quality of life. As a limitation to this review, all studies included in the current study were cross-sectional. This limits the causal conclusion between prediabetes and associated factors because confounding variables may affect the outcome variable. Moreover, only articles published in English were considered for this subregional review.

## 5 Conclusion

The prevalence of prediabetes in the adult East African population was high. This study revealed that old age, high BMI and hypertension were associated with prediabetes. The study findings suggest that public health policymakers should pay attention to the magnitude of prediabetes and develop a system for community screening targeted at those with obesity, hypertension, and old age. A health policy that focuses on prevention and control strategies is urgently needed to reduce the prevalence of prediabetes and subsequent type 2 diabetes through lifestyle interventions.

## Patients and public involvement

Not applicable.

## Data availability statement

Data will be made available on request.

## Consent for publication

All authors provided written informed consent to publish this study.

## Funding

Not applicable.

## Additional files

Not applicable.

## CRediT authorship contribution statement

**Daniel Asmelash:** Conceptualization, Data curation, Formal analysis, Funding acquisition, Investigation, Methodology, Project administration, Resources, Software, Supervision, Validation, Visualization, Writing – original draft, Writing – review & editing. **Getachew Mesfin Bambo:** Data curation, Formal analysis, Funding acquisition, Investigation, Methodology, Project administration, Resources, Software, Supervision, Validation, Visualization, Writing – original draft, Writing – review & editing. **Samuel Sahile:** Data curation, Formal analysis, Funding acquisition, Investigation, Methodology, Project administration, Resources, Software, Supervision, Validation, Visualization, Writing – original draft, Writing – review & editing. **Yemane Asmelash:** Data curation, Formal analysis, Funding acquisition, Investigation, Methodology, Project administration, Resources, Software, Validation, Visualization, Writing – original draft, Writing – review & editing.

## Declaration of competing interest

The authors declare that they have no known competing financial interests or personal relationships that could have appeared to influence the work reported in this paper.

## List of abbreviations

ADA: American diabetes association
BMI: Body mass index
DM: Diabetes Mellitus
T2DM: Type 2 Diabetes mellitus
UDM: Glycated hemoglobin (HbA1c)
FPG: Fasting blood glucose
CI: Confidence interval
IFG: impaired fasting glucose
OR: Odds ratio
WHO: World Health Organization
