# Supplementary material for: Prevalence and associated factors of prediabetes in adult East African population: A systematic review and meta-analysis
Source: Heliyon. 2023 Oct 20;9(11):e21286. doi: 10.1016/j.heliyon.2023.e21286 (PMC10623273; doi:10.1016/j.heliyon.2023.e21286)
Supplement: Multimedia component 1 [file mmc1.docx]

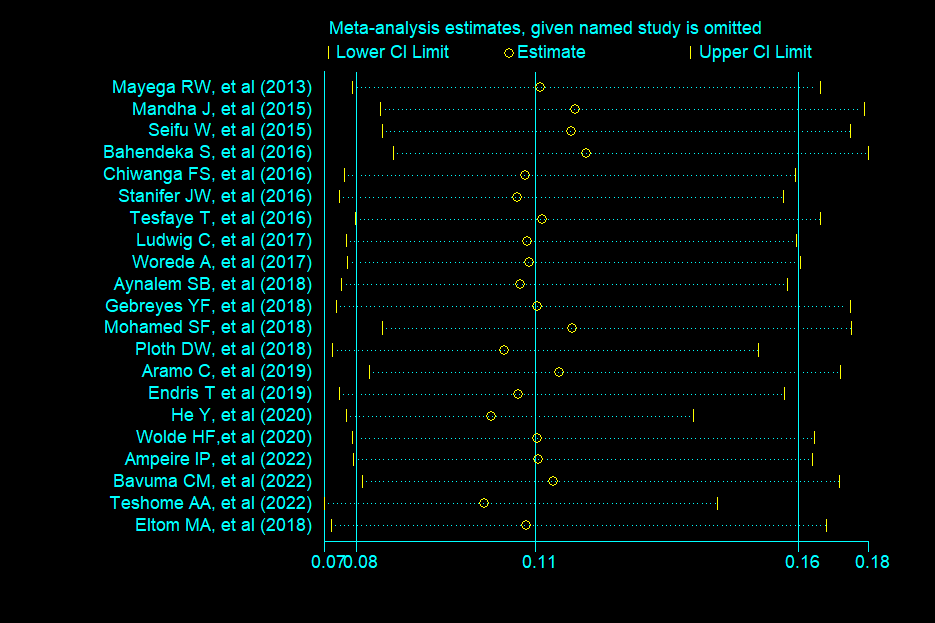


Figure 1. Sensitivity analysis of the prevalence of prediabetes

Table 1. Meta regression of publication year with the prevalence of prediabetes

| Prev. | Coef. Std. Err. t P>t [95% Conf. Interval] |
| --- | --- |
| year | 1.791774 .9308254 1.92 0.069 -.1564656 3.740014 |
